# Clinicopathological characteristics and outcomes of synchronous renal cell carcinoma and urothelial carcinoma: A population-based analysis

**DOI:** 10.3389/fpubh.2022.994351

**Published:** 2022-10-31

**Authors:** Kan Wu, Xu Liu, Yaohui Wang, Xianding Wang, Xiang Li

**Affiliations:** ^1^Department of Urology, Institute of Urology, West China Hospital, Sichuan University, Chengdu, China; ^2^Breast Disease Center, Cancer Center, West China Hospital, Sichuan University, Chengdu, China

**Keywords:** renal cell carcinoma, urothelial carcinoma, concurrent tumor, survival outcome, Surveillance Epidemiology and End Results (SEER) database

## Abstract

**Background:**

To better understand the characteristics, and survival outcomes of synchronous renal cell carcinoma (RCC) and urothelial carcinoma (UC), we described and analyzed the clinical features, factors, and prognosis of patients with synchronous RCC and UC using a large population-based database.

**Methods:**

Within the Surveillance, Epidemiology, and End Results (SEER) database (2004–2016), we identified patient with concurrent RCC and UC at initial diagnosis. Their clinicopathological features and prognosis were evaluated. A logistic regression model was used to examine risk factors for the occurrence of concomitant RCC and UC, and Kaplan-Meier survival curves were used to estimate overall survival.

**Results:**

A total of 61,454 RCC patients were identified from the SEER database, 704 (1.1%) patients presented with synchronous RCC and UC. Among these patients, concurrent bladder tumors (566/704) are more common. Subsequently, subgroup analysis based on the location of UC indicated that patients with concurrent RCC and upper tract urothelial carcinoma (UTUC) had unfavorable UC characteristics (higher tumor stage and grade), compared with patients with concomitant bladder cancer. An increased risk of concurrent UC was observed among older age, male sex, and white race. Meanwhile, papillary RCC histology [odds ratio (OR) 3.23; 95% confidence interval (CI) 2.13–4.90], and smaller tumor (OR 6.63; 95% CI 4.46–9.87) were independent risk factors for concomitant UTUC. In addition, we found that synchronous RCC and UTUC was associated with worse survival by using Kaplan-Meier and multivariable analysis [hazard ratio (HR) 2.36, 95% CI 1.89–2.95]. However, concomitant bladder cancer did not affect survival outcomes of patients with RCC (HR 1.00, 95% CI 0.86–1.17).

**Conclusion:**

We found that synchronous concurrent RCC and UC is relatively uncommon and mostly located in the bladder. Older age, male sex, and white race increase the risk of synchronous RCC and UC. Meanwhile, patients with papillary RCC histology, and smaller tumors are more likely to have concomitant RCC and UTUC. Furthermore, our findings suggest that synchronous RCC and UTUC has a worse prognosis, while, concomitant bladder tumor did not affect the oncological outcomes of RCC.

## Introduction

Renal cell carcinoma (RCC) represents about 3.8% of all new malignancies, leading to ~ 76,080 new cases of RCC in the United States in 2021 (1). Urothelial carcinoma (UC) is the fourth most commonly diagnosed cancer in men in developed countries (1), which can be located in the lower (bladder) or the upper (renal pelvis and ureters) urinary tract. Bladder cancer (BC) accounts for 90–95% of all urothelial cancers and is the most common urinary tract neoplasm (2, 3). However, upper tract urothelial carcinoma (UTUC) is relatively uncommon and represents only 5–10% of UCs (4). The incidence of these tumors has risen over the past few decades as detection has improved (5, 6). Particularly, both malignancies, RCC and UC, share some common etiological risk factors, such as exposure to smoking tobacco, and aristolochic acid (7–9). Thus, theoretically, an increase in the prevalence of genitourinary malignancies would lead to a reasonable increase in the diagnosis of synchronous tumors. We need a better understanding of the prevalence, characteristics, and prognostic outcomes of synchronous RCC and UC.

Most studies on this topic, however, are limited by sample size, small series, and great heterogeneity of results (10). At present, only about 60 cases of synchronous renal tumors and renal pelvic cancers have been reported in the English literature (11–19). In addition, the characteristics and outcomes of concurrent RCC and UC are still unclear. Specifically, a review of 47 cases of simultaneous RCC and UC showed no effect of concomitant UC on overall prognosis (16). Conversely, some case series suggest that synchronous tumors represent a more aggressive feature, and risk factor of survival outcome (11, 14). Taken together, due to the rarity of concurrent cancer, it remains uncertain if the simultaneous RCC and UC would adversely affect overall prognosis, compared with isolated ones.

To date, no larger series has investigated the clinical features, risk factors, and potential oncological effects of concurrent RCC and UC. Therefore, in this study, we sought to describe the occurrence pattern and characteristics of simultaneous RCC and UC based on nationally representative Surveillance, Epidemiology, and End Results (SEER) data. We also attempted to examine the risk factors, and oncological outcomes of patients diagnosed with synchronous RCC and UC.

## Methods

### Study population

We identified patients with synchronous RCC and UC from the National Cancer Institute's SEER database between 2004 and 2016. Patients with concurrent RCC and UC at the first diagnosis or who had a new urothelial cancer within 3 months of RCC diagnosis were considered to have synchronous RCC and UC.

Based on International Classification of Diseases for Oncology (3rd edition) codes (20), patients with primary site and histologic codes labeled as “Renal: RCC” and/or “Renal pelvis, Ureter, or Bladder: transitional cell carcinoma” were included in this study. Only microscopically confirmed cases were included. Patients < 18 years at initial diagnosis were excluded. Patients with bilateral RCC, distant disease, follow-up <3 months or unknown survival status were also excluded.

From the SEER database, 61,454 patients with RCC were eligible for inclusion, all patients were divided into two groups: Only RCC vs. synchronous RCC and UC. While UTUC share morphology and histological appearance with BC, recent molecular investigations suggest that UTUC and BC represent two distinct disease entities (21, 22). Therefore, subgroup analysis was performed based on the location of UC (bladder, renal pelvis, and ureter).

### Clinical variables

Demographic variables included sex, patient age at the first diagnosis, marital status, and race. Clinicopathological covariates included tumor laterality, tumor size, stage, pathology grade, and histologic subtype. Overall survival (OS) was the primary outcome in the current study. The SEER database does not provide information on the number of pathologists at the time of diagnosis. Presently, for tumor stage that recorded in the SEER system, two methods commonly used to determine stage are AJCC and SEER historic. The AJCC method is more commonly used in the clinical settings, while SEER has standardized and simplified staging to ensure consistent definitions over time. In this study, we used the AJCC 6th edition TNM staging system. In addition, the SEER Program registries record the pathology grade of RCC and UC as “Grade I: Well–differentiated”, “Grade II: Moderately differentiated”, “Grade III: Poorly differentiated” or “Grade IV: Undifferentiated; anaplastic” and these categorizations were used in this study. Age at diagnosis was categorized into two groups: <65, and ≥65 years. Tumor stage was classified as stage I/II and stage III/IV. Pathology grade was categorized as I/II well-differentiated, III/IV poor to undifferentiated, and unknown. The histologic subtype of RCC was categorized into clear cell, papillary, chromophobe, other, and RCC not otherwise specified. Tumor size was categorized as <3.0 and ≥3 cm. AJCC 7th edition.

### Statistical analysis

In this study, only age at the first diagnosis and tumor size were continuous variables, and required the normality tests. By using Kolmogorov-Smirnov tests (Suitable for large samples > 5000), we found that neither age at diagnosis (*P* < 0.001) nor tumor size (*P* < 0.001) followed a normal distribution. Therefore, Kruskal-Wallis non–parametric test was used to compare differences in continuous variables between patient groups. Chi-square tests were used to assess the differences in categorical variables between patient groups. A univariate and multivariate logistic regression model was created to identify risk factors associated with concomitant RCC and UC, and expressed as odd ratio (OR) with 95% confidence interval (CI). Kaplan–Meier survival curves were applied to estimate and compare differences in OS between patients with only RCC and patients with synchronous RCC and UC. Multivariable Cox regression analysis model was then performed to examine the effects of concomitant UC on survival.

P < 0.05 was considered statistically significant and all *P*-values were two-sided. Kaplan-Meier survival analysis followed by log-rank test was performed using GraphPad Prism version 5.00 for Windows, GraphPad Software, San Diego, California United States, www.graphpad.com. Other statistical analyses were performed using SPSS software version 23.0(IBM Corp. Released 2015. IBM SPSS Statistics for Windows, Version 23.0. Armonk, NY: IBM Corp).

## Results

### Patient characteristics

A total of 61,454 patients were diagnosed with RCC, including 704 patients (1.1%) presenting with synchronous RCC and UC (Table 1). The median age of all patients was 61 years (interquartile range [IQR], 52–69 years). The median size of the primary RCC was 4.1 cm (IQR, 2.7–6.5 cm). Men, and women accounted for 62.3%, and 37.7% of the population, respectively. White and other race represented 81.2% and 18.8% of cases, respectively.

**Table 1:** Patient characteristics^a^.

	**Synchronous RCC and UC**			
**Characteristic**	**No (*n* = 60,750)**	**BC (*n* = 566)**	**UTUC (*n* = 138)**	***P***
Age, years (median, IQR)	60 (51–69)	69 (61–75)	72 (66–78)	< 0.001
< 65	37,679 (62.0)	188 (33.2)	30 (21.7)	< 0.001
≥65	23,071 (38.0)	378 (66.8)	108 (78.3)	
**Sex (** ***n*** **, %)**				< 0.001
Men	37,734 (62.1)	466 (82.3)	115 (83.3)	
Women	23,016 (37.9)	100 (17.7)	23 (16.7)	
**Race (** ***n*** **, %)**				< 0.001
White	49,277 (81.1)	498 (88.0)	122 (88.4)	
Other	11,473 (18.9)	68 (12.0)	16 (11.6)	
**Marital status (** ***n*** **, %)**				0.149
Married	36,831 (60.6)	356 (62.9)	87 (63.0)	
Not married	20,823 (34.3)	174 (30.7)	48 (34.8)	
Unknown	3,096 (5.1)	36 (6.4)	3 (2.2)	
**Laterality (** ***n*** **, %)**				0.034
Left	29,821 (49.1)	248 (43.8)	72 (52.2)	
Right	30,929 (50.9)	318 (56.2)	66 (47.8)	
**Stage (** ***n*** **, %)**				0.037
I/II	47,603 (78.4)	459 (81.1)	116 (84.1)	
III/IV	11,603 (19.1)	88 (15.5)	17 (12.3)	
Unknown	1,544 (2.5)	19 (3.4)	5 (3.6)	
**Histology (** ***n*** **, %)**				< 0.001
RCC, NOS	9,478 (15.6)	94 (16.6)	30 (21.7)	
Clear cell RCC	37,512 (61.7)	328 (58.0)	51 (37.0)	
Papillary RCC	7,554 (12.4)	93 (16.4)	45 (32.6)	
Chromophobe RCC	3,925 (6.5)	27 (4.8)	7 (5.1)	
Other RCC	2,281 (3.8)	24 (4.2)	5 (3.6)	
**Pathology grade (** ***n*** **, %)**				0.031
I/II well-differentiated	33,617 (55.3)	312 (55.2)	77 (55.8)	
III/IV poor to undifferentiated	17,794 (29.3)	144 (25.4)	35 (25.4)	
Unknown	9,339 (15.4)	110 (19.4)	26 (18.8)	
Tumor size, cm (median, IQR)	4.1 (2.7–6.5)	3.8 (2.7–6.0)	1.5 (0.8–3.1)	< 0.001
< 3	16,763 (27.6)	153 (27.0)	93 (67.4)	< 0.001
≥3	43,079 (70.9)	400 (70.7)	39 (28.3)	
Unknown	908 (1.5)	13 (2.3)	6 4.3()	

RCC, renal cell carcinoma; UC, urothelial carcinoma; BC, bladder cancer; UTUC, upper urinary urothelial carcinoma; IQR, interquartile range; NOS, not specifically.

^a^Kruskal-Wallis non–parametric test was used for comparison of continuity variables between groups, while chi-square tests were used for categorical variables.

Table 1 shows the baseline clinical and pathological characteristics of the study population. Among the 704 patients with synchronous RCC and UC, 566 (80.4%) patients were diagnosed with RCC concurrent with BC, 138 (19.6%) patients were concurrent RCC and UTUC. Concurrent tumors were more common among men and among whites (Table 1). In addition, those with synchronous RCC and UC were diagnosed at an older age, were smaller tumor size, and were of papillary RCC histology.

On analysis of concurrent UTUC, 126 (17.9%) patients were concurrent RCC and ipsilateral UTUC (renal pelvic = 80, ureter = 46), 9 cases were RCC and concomitant contralateral UTUC (renal pelvic = 8, ureter = 1), two cases were RCC concurrent with ipsilateral renal pelvic carcinoma and bladder tumor, one patient was RCC and ipsilateral ureter carcinoma and bladder cancer.

All patients with concurrent RCC and UC were further classified into three subgroups: bladder cancer, renal pelvic and ureter carcinoma (Supplementary Table). Concurrent ureter carcinomas were more common among patients with older age (*P* = 0.001). Analysing the characteristics of primary RCC, patients with concurrent UTUC more commonly had papillary subtype of RCC, compared with patients with concomitant bladder tumor. The majority of patients underwent surgical resection of kidney cancer. For analyzing the characteristics of concomitant UC (Supplementary Table), most patients with concurrent bladder cancer had low tumor stage, compared with patients with renal pelvic and ureter carcinoma (*P* < 0.001). In addition, patients with synchronous RCC and BC had primary tumors were small and low pathology grade (*P* < 0.001). Total surgical resection of primary UC was more performed in patients who diagnosed with concurrent UTUC, compared with patients with concomitant BC (*P* < 0.001).

### Risk factors of synchronous renal cell carcinoma and urothelial carcinoma

A univariate and multivariate logistic regression model was performed to evaluate risk of concomitant RCC and UC (Table 2). For synchronous RCC and BC, in the univariate analysis, at initial diagnosis (*P* < 0.001), sex (*P* < 0.001), race (*P* < 0.001), tumor laterality (*P* = 0.013), tumor stage (*P* = 0.039), and histological type of RCC (*P* = 0.020) were correlated with the occurrence of RCC concurrent with bladder tumor. By multivariate analysis, older age (≥ 65 years vs. < 65 years, OR 3.34; 95% CI 2.79–3.99), and male sex (OR 3.05; 95% CI 2.44–3.81) were associated with significantly increased risk of synchronous RCC and BC. While, non-Caucasians (OR 0.63; 95% CI 0.48–0.81), and higher tumor stage (OR 0.66; 95% CI 0.52–0.83) were associated with significantly decreased risk of concurrent RCC and bladder tumor.

**Table 2:** Logistic regression analysis of risk factors for synchronous RCC and UC^a^.

	**BC**			**UTUC**		
	**Univariate**	**Multivariate**		**Univariate**	**Multivariate**	
**Characteristic**	***P* value**	**OR (95% CI)**	***P***	***P* value**	**OR (95% CI)**	***P***
Age	< 0.001*			< 0.001*		
< 65		REF			REF	
≥65		3.34 (2.79–3.99)	< 0.001*		6.23 (4.11–9.44)	< 0.001*
Sex	< 0.001*			< 0.001*		
Women		REF			REF	
Men		3.05 (2.44–3.81)	< 0.001*		3.38 (2.11–5.42)	< 0.001*
Race	< 0.001*			0.029*		
White		REF			REF	
Other		0.63 (0.48–0.81)	< 0.001*		0.51 (0.29–0.89)	0.017*
Marital status	0.118			0.293		
Married						
Not married						
Laterality	0.013*			0.469		
Left		REF				
Right		1.19 (1.00–1.41)	0.050			
Stage	0.039*			0.048*		
I/II		REF			REF	
III/IV		0.66 (0.52–0.83)	< 0.001*		1.02 (0.58–1.77)	0.957
Histology	0.020*			< 0.001*		
Clear cell RCC		REF			REF	
Papillary RCC		1.21 (0.95–1.54)	0.118		3.23 (2.13–4.90)	< 0.001*
Chromophobe RCC		0.88 (0.60–1.31)	0.540		1.22 (0.52–2.85)	0.651
Other RCC		1.27 (0.84–1.94)	0.260		1.85 (0.73–4.69)	0.192
RCC, NOS		1.08 (0.85–1.38)	0.510		2.08 (1.29–3.33)	0.002*
Pathology grade	0.175			0.455		
I/II						
III/IV						
Tumor size	0.857			< 0.001*		
≥3 cm					REF	
< 3 cm					6.63 (4.46–9.87)	< 0.001*

RCC, renal cell carcinoma; UC, urothelial carcinoma; BC, bladder cancer; UTUC, upper urinary urothelial carcinoma; OR, odds ratio; CI, confidence interval; REF, reference; NOS, not specifically.

^a^First, univariate analysis (chi-square test) was used to identify variables that may be meaningful, and then variables with *p* < 0.05 in univariate analysis were included in the multivariate logistic regression model.

^*^Statistically significant.

Furthermore, we used the logistic regression model to evaluate risk of synchronous RCC and UTUC (Table 2). In the univariate analysis, age at primary diagnosis (*P* < 0.001), gender (*P* < 0.001), ethnicity (*P* = 0.029), tumor stage (*P* = 0.048), and histological type of RCC (*P* < 0.001), and tumor size (*P* < 0.001) were associated with the occurrence of concurrent disease. In multivariable analysis, older age at primary diagnosis (≥ 65 years vs. < 65 years, OR 6.23; 95% CI 4.11–9.44), male sex (OR 3.38; 95% CI 2.11–5.42), papillary RCC histology (OR 3.23; 95% CI 2.13–4.90), and smaller tumor size (< 3 cm vs. ≥ 3 cm, OR 6.63; 95% CI 4.46–9.87) were independent risk factors for RCC concurrent with UTUC. Non-Caucasians was associated with a reduced risk of concomitant RCC and UTUC (OR 0.51; 95% CI 0.29–0.89).

### Treatment and survival outcome of synchronous cancer

Different operative approaches were performed according to the type of concurrent tumor. For patients with synchronous RCC and bladder cancer (566 patients), 55.1% (312/566) underwent radical nephrectomy, 32% (181/566) received partial nephrectomy, 3.4% (19/566) were treated with tumor local excision, and 7.1% (40/566) did not receive surgical treatment. For patients diagnosed with RCC and ipsilateral UTUC (126 patients), most patients (104/126: renal pelvic = 66/80, ureter = 38/46) underwent radical nephroureterectomy (RNU). Three patients diagnosed with RCC and contralateral renal pelvic carcinoma underwent partial nephrectomy and contralateral RNU. One patient with RCC and contralateral ureter tumor underwent partial nephrectomy and contralateral ureteroureterostomy.

**Figure 1:**
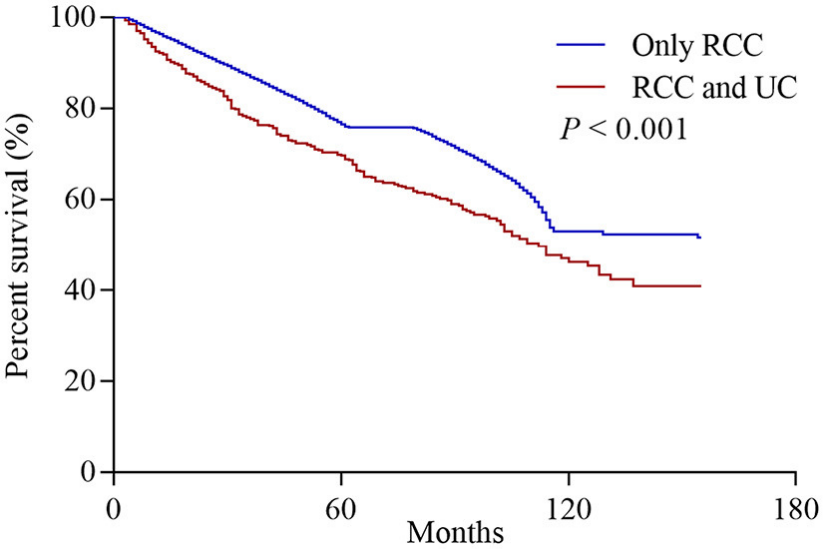
Overall survival of patients who initially presented with solitary renal cell carcinoma (RCC) or synchronous concurrent RCC and urothelial carcinomas (UC).

**Figure 2:**
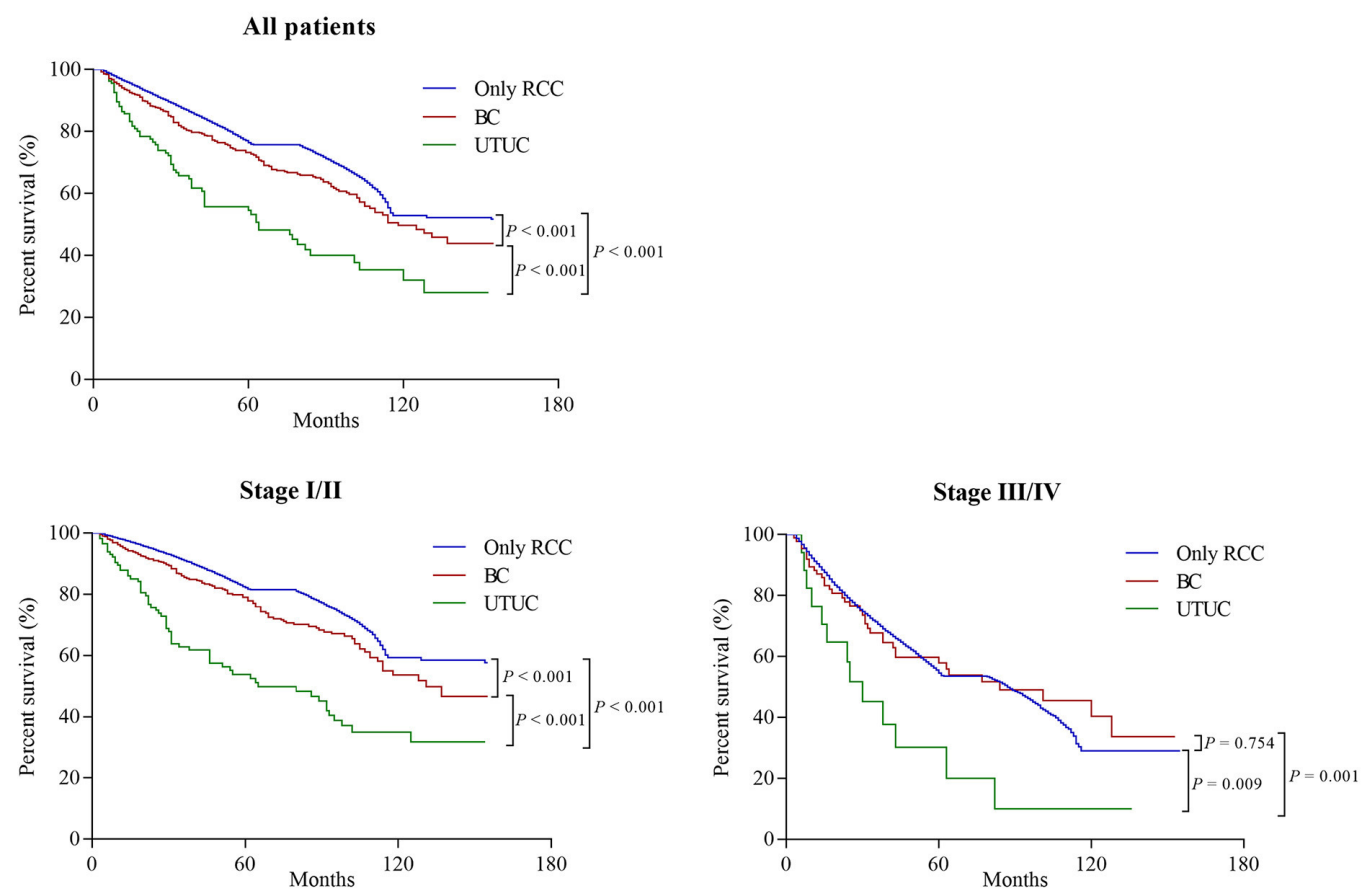
Overall survival of patients who initially presented with solitary renal cell carcinoma (RCC) or synchronous RCC and urothelial carcinomas (UC) based on the location of UC (Bladder cancer [BC] and upper tract urothelial carcinoma [UTUC]), stratified by tumor stage at diagnosis.

Among all initially unilateral RCC patients, 11,966 (19.5%) patients died during the follow-up (median time: 39 months). As shown in Figure 1, compared to patients with only RCC, synchronous RCC and UC patients had a worse survival (5-year OS rate: 76.5% vs. 69.6%, *P* < 0.001). In subgroup analysis according to anatomical location of concurrent tumors, the 5-years OS rates for patients with only RCC, concurrent BC, and UTUC were 76.5, 75, and 49.0%, respectively (*P* < 0.001, Figure 2 and Table 3). Both the survival analysis and multivariable Cox regression model showed that patients with synchronous RCC and UTUC had significantly shorter OS than those with only RCC [hazard ratio (HR) 2.36, 95% CI 1.89–2.95; Table 3]. Subgroup analysis also showed that the diagnosis of RCC concurrent with UTUC was associated with poor survival outcomes of patients regardless of tumor stage (Figure 2, Table 3). However, concurrent bladder cancer did not increase the mortality risk of patients with RCC in the multivariable model.

**Table 3:** Overall survival according to multivariate analysis in different groups^a^.

	**Synchronous RCC and UC**	**5-year overall survival rate, %**	**HR (95% CI)**	***P* value**
All (*n* = 61,454)	No (*n* = 60,750)	76.5	Reference	
	BC (*n* = 566)	75.0	1.00 (0.86–1.17)	0.982
	UTUC (*n* = 138)	49.0	2.36 (1.89–2.95)	< 0.001
Stage I/II (*n* = 48,178)	No (*n* = 47,603)	82.2	Reference	
	BC (*n* = 459)	79.1	1.05 (0.88–1.26)	0.593
	UTUC (*n* = 116)	54.1	2.12 (1.65–2.73)	< 0.001
Stage III/IV (*n* = 11,708)	No (*n* = 11,603)	54.5	Reference	
	BC (*n* = 88)	57.9	0.82 (0.60–1.13)	0.239
	UTUC (*n* = 17)	22.0	2.19 (1.27–3.78)	0.005

RCC, renal cell carcinoma; UC, urothelial carcinoma; BC, bladder cancer; UTUC, upper urinary urothelial carcinoma; HR, hazard ratio; CI, confidence interval.

^a^HR in multivariate Cox analysis were adjusted for age, sex, stage, tumor grade, tumor histology, the mode of surgery.

## Discussion

In this largest population cohort of more than 60 000 patients with RCC, we found that a total of 704 patients (1.1%) were initially diagnosed with synchronous RCC and UC. Among these patients, concurrent RCC and bladder tumors (566/704) are more common. Patients with older age at primary diagnosis, and male had a higher risk of synchronous RCC and UC. While, non-Caucasian was associated with a reduced risk of concurrent RCC and UC. Interestingly, papillary RCC histology, and smaller tumors increase one's likelihood of having concomitant RCC and UTUC. Although the overall incidence is relatively low (1.1%), the risk is significantly increased in certain patients. Other new findings based on this cohort include that synchronous RCC and UTUC was associated with a poor survival, compared with patients with a solitary renal tumor. However, concurrent bladder cancer did not affect the oncological outcomes of patients with RCC.

The diagnosis of multiple primary malignancy in genitourinary system is relatively uncommon. The first case with synchronous RCC and UC was described by Graves and Templeton in 1921 (23). Studies focusing on concurrent RCC and UC are mostly case reports or small case series in the literature. Due to the rarity of this disease and the limited studies, the tumor characteristics of both entities were not well–assessed, especially for concurrent bladder tumor. In this study, we retrospectively evaluated the characteristics of 704 patients who presented with synchronous RCC and UC based on a population-based cohort. As far as we know, this is the largest cohort of patients with synchronous RCC and UC ever reported in English-language literature. Our current findings indicated that the proportion of papillary RCC histology was significantly increased in patients with concurrent RCC and UC, especially in synchronous UTUC, compared with the histological type of solitary renal cancer (the percentage of papillary histology in solitary RCC: 12.4%, bladder tumor: 16.4%, pelvis tumor: 28.9%, ureter tumor: 39.6%). Also interesting is that subgroup analysis based on the location of UC showed that concurrent RCC and UTUC had unfavorable UC characteristics, such as higher pathological grade and stage. Understanding the tumor characteristics and potential oncological effects of synchronous RCC and UC, is important to develop appropriate surveillance and management for these patients.

The gold-standard management of UTUC is RNU (24, 25), so it appears that the best treatment option for synchronous ipsilateral RCC and UTUC should also be RNU. This consensus was confirmed in our study, where most cases with synchronous ipsilateral RCC and UTUC in this cohort received RNU. However, due to the low incidence of synchronous RCC and contralateral ureter carcinoma, opinions on the treatment of patients with RCC and contralateral UTUC, are not unified, and there is no general clinical guideline or consensus. We believe that all factors, such as the biological characteristics of bilateral tumors originating from different histogenesis, bilateral renal function, and the patient's quality of life, should be comprehensively considered to formulate an individualized management plan. Likewise, only a few studies describe treatment modalities for RCC and concurrent bladder tumor (11, 26). However, in this study, we found that combined RCC and bladder tumor were more common. Therefore, it is necessary to pay attention to the possible occurrence and treatment of synchronous RCC and bladder tumor.

Previous studies have emphasized poor survival outcomes in patients with concurrent RCC and UC, but a literature review of 47 cases showed that the occurrence of concurrent RCC and UC did not affect the patient's oncological prognosis (16). It is worth noting, however, that the patient number of published studies is small and usually comes from the referral centers. Based on the largest population cohort, we found that concomitant bladder tumor does not represent a risk factor for survival outcomes of RCC. While, concurrent tumors located in the renal pelvis or ureter can would compromise poor oncological outcomes. Therefore, our results suggest that the prognosis of patients with synchronous dual malignancies may be most affected by the more aggressive one, as we found that UC located in the upper urinary tract had higher tumor stage, grade, and worse prognosis than UC located in the bladder. It is worth noting that compared with the BC group, patients in the UTUC group have advanced age at the first diagnosis, as a common risk factor of unfavorable outcomes. In addition to clinicopathological and histological aspects, the molecular alterations of UTUC that differ from BC may have a significant impact on patient outcomes (27). For example, UTUC samples harbored high prevalence of FGFR3 alterations, which could potentially be responsible for immune cell depleted phenotype and inferior oncological outcomes (28).

The main advantage of this population-based study is its large sample size, which allowed us to perform statistical analysis on a relatively large cohort of synchronous RCC and UC, while avoiding the effect of patient selection bias from referral centers. Therefore, the characteristics and survival outcomes of concurrent RCC and UC we describe are somewhat representative. However, there are some limitations of our study, including the lack of detailed information on the multifocality of the lesions, and the lack of data on adjuvant therapy. Furthermore, we do not have detailed information on patient comorbidities, performance status, and environmental factors (exposure to tobacco, chemical carcinogen, and aristolochic acid), which are not available in SEER, which may represent potential confounders. Additionally, because only pathologically confirmed disease was included in this study, those patients without pathological confirmation of diagnosis were not collected, which may lead to underreporting of synchronous RCC and UC.

## Conclusions

In conclusion, concurrent UC is relatively uncommon in renal cancer (1.1%), and it is mostly located in the bladder. However, specific characteristics that increase the risk of synchronous RCC and UC include older age, male sex, and white race. Surprisingly, we found that papillary RCC histology, and smaller tumors were also associated with the occurrence of concomitant RCC and UTUC. Furthermore, our data suggest that concomitant bladder tumor does not affect the oncological outcomes of patients with RCC, but concomitant UTUC has a worse prognosis compared with patients with solitary RCC. Consequently, our findings provide reasonable evidence that clinicians should consider the biological characteristics of each tumor to develop an individualized treatment strategy.

## Data availability statement

The original contributions presented in the study are included in the article/Supplementary material, further inquiries can be directed to the corresponding authors.

## Ethics statement

Ethical approval was not provided for this Study on Human Participants Because We Signed the SEER Research Data Agreement and Further Searched for Data According to the Approved Guidelines. The SEER data were open available and patients' records are anonymous, therefore, this study was deemed exempt from review by our institutional review board. Written informed consent for participation was not required for this study in accordance with the national legislation and the institutional requirements.

## Author contributions

KW, XLiu, and YW collected, analyzed, interpreted the data, and prepared the initial draft of manuscript. XW analyzed and interpreted the data, analyzed the statistics, and critically reviewed the manuscript. XW and XLi designed the study, and critically reviewed the manuscript, have full access to all the data in the study, and final responsibility for the decision to submit for publication. All authors contributed to the article and approved the submitted version.

## Funding

This work was supported by the 1.3.5 project for disciplines of excellence, West China Hospital, Sichuan University.

## Conflict of interest

The authors declare that the research was conducted in the absence of any commercial or financial relationships that could be construed as a potential conflict of interest.

## Publisher's note

All claims expressed in this article are solely those of the authors and do not necessarily represent those of their affiliated organizations, or those of the publisher, the editors and the reviewers. Any product that may be evaluated in this article, or claim that may be made by its manufacturer, is not guaranteed or endorsed by the publisher.
