# Supplementary material for: Clinicopathological characteristics and outcomes of synchronous renal cell carcinoma and urothelial carcinoma: A population-based analysis
Source: Front Public Health. 2022 Oct 31;10:994351. doi: 10.3389/fpubh.2022.994351 (PMC9659638; doi:10.3389/fpubh.2022.994351)
Supplement: Supplementary file 1 [file Table_1.DOCX]

| **Supplementary Table.** The characteristics of patients with synchronous RCC and UC by anatomical location^a^ | | | | |
| --- | --- | --- | --- | --- |
| Characteristic | Bladder  (n = 566) | Pelvis  (n = 90) | Ureter  (n = 48) | *P* |
| Age, y, median | 69 | 70 | 74 | 0.001 |
| Sex |  |  |  | 0.618 |
| Men | 466 (82.3) | 77 (85.6) | 38 (79.2) |  |
| Women | 100 (17.7) | 13 (14.4) | 10 (20.8) |  |
| **RCC** | | | | |
| Stage |  |  |  | 0.194 |
| I/II | 459 (81.1) | 76 (84.4) | 40 (83.3) |  |
| III/IV | 88 (15.5) | 13 (14.4) | 4 (8.3) |  |
| Unknown | 19 (3.4) | 1 (1.1) | 4 (8.3) |  |
| Surgery for RCC |  |  |  | < 0.001 |
| No | 40 (7.1) | 4 (4.4) | 0 (0.0) |  |
| Tumor local excision | 19 (3.4) | 2 (2.2) | 0 (0.0) |  |
| Partial nephrectomy | 181 (32.0) | 11 (12.2) | 2 (4.2) |  |
| Radical nephrectomy | 312 (55.1) | 71 (78.9)) | 43 (89.6)) |  |
| Nephrectomy, NOS | 14 (2.5) | 2 (2.2) | 3 (6.2) |  |
| Histology |  |  |  | < 0.001 |
| RCC, NOS | 94 (16.6) | 20 (22.2) | 10 (20.8) |  |
| Clear cell RCC | 328 (58.0) | 34 (37.8) | 17 (35.4) |  |
| Chromophobe RCC | 27 (4.8) | 6 (6.7) | 1 (2.1) |  |
| Papillary RCC | 93 (16.4) | 26 (28.9) | 19 (39.6) |  |
| Other RCC | 24 (4.2) | 4 (4.4) | 1 (2.1) |  |
| Pathology grade |  |  |  | 0.928 |
| I/II well-differentiated | 312 (55.2) | 51 (56.7) | 26 (54.2) |  |
| III/IV poor to undifferentiated | 144 (25.4) | 24 (26.7) | 11 (22.9) |  |
| Unknown | 110 (19.4) | 15 (16.6) | 11 (22.9) |  |
| Tumor size, cm, median | 3.8 | 2.0 | 1.0 | < 0.001 |
| **UC** |  |  |  |  |
| Stage |  |  |  | < 0.001 |
| I/II | 528 (93.3) | 42 (46.7) | 33 (68.8) |  |
| III/IV | 24 (4.2) | 43 (47.8) | 15 (31.2) |  |
| Unknown | 14 (2.5) | 5 (5.6) | 0 (0.0) |  |
| Surgery for UC |  |  |  | < 0.001 |
| No | 27 (4.8) | 5 (5.6) | 2 (4.2) |  |
| Tumor local excision | 8 (1.4) | 3 (3.3) | 0 (0.0) |  |
| Partial surgery | 493 (87.1) | 8 (8.9) | 5 (10.4) |  |
| Total surgery | 15 (2.7) | 70 (77.8) | 38 (79.2) |  |
| Unknown | 23 (4.1) | 4 (4.4) | 3 (6.2) |  |
| Pathology grade |  |  |  | < 0.001 |
| I/II well-differentiated | 252 (44.5) | 18 (20.0) | 12 (25.0) |  |
| III/IV poor to undifferentiated | 195 (34.5) | 62 (68.9) | 31 (64.6) |  |
| Unknown | 119 (21.0) | 10 (11.1) | 5 (10.4) |  |
| Tumor size, cm, median | 2.4 | 3.5 | 2.5 | < 0.001 |
| RCC, renal cell carcinoma; UC, urothelial carcinoma; NOS, not specifically.  ^a^ Kruskal-Wallis nonparametric test was used for comparison of continuity variables between groups, while chi-square tests were used for categorical variables. | | | | |
